# Study on the gut microbiota, HPA, and cytokine levels in infantile spasms

**DOI:** 10.3389/fimmu.2024.1442677

**Published:** 2024-10-10

**Authors:** Jiajia You, Li Liu, Xiongfeng Pan, Liwen Wu, Lihong Tan, Changci Zhou, Siwei Fang, Zhenghui Xiao, Jun Qiu

**Affiliations:** ^1^ The School of Pediatrics, Hengyang Medical School, University of South China (Hunan Children’s Hospital), Hengyang, China; ^2^ Pediatrics Research Institute of Hunan Province, Hunan Children’s Hospital, Changsha, China; ^3^ Department of Neurology, Hunan Children’s Hospital, Changsha, China; ^4^ Department of Emergency Center, Hunan Children’s Hospital, Changsha, China

**Keywords:** gut microbiota, infantile spasm, inflammatory cytokines, HPA axis hormones, correlations

## Abstract

**Objective:**

The mechanisms driving the progression of infantile spasms are not well understood. We aimed to investigate the changes and correlations of the gut microbiota, the hypothalamus–pituitary–adrenal (HPA) axis hormones, and the inflammatory cytokines in children with infantile spasms before and after treatment in order to provide a reference for future pathogenesis research.

**Methods:**

Children with infantile spasms who were admitted to our hospital were recruited into the case group. The case group was divided into the pre-treatment group (group A, *n* = 14), the 2 weeks after treatment group (group B), and the 1 month after treatment group (group C). On the other hand, healthy children with the same sex ratio as the case group were recruited into the control group (group D, *n* = 14). Three stool and blood samples were collected before treatment, 2 weeks after treatment, and 1 month after treatment. The serum samples were analyzed using cytometric bead array (CBA), enzyme-linked immunosorbent assay (ELISA), and chemiluminescent immunoassay (CLIA) to measure the levels of HPA axis hormones and inflammatory cytokines. The collected stool samples were sequenced using 16S rDNA.

**Results:**

The pre-treatment group demonstrated elevated levels of corticotropin-releasing hormone (CRH), interleukin 2 (IL-2), IL-4, IL-6, and IL-17α, which decreased with treatment. The level of CRH was lower in the effective group than that in the ineffective group. *Sutterellaceae* was lower in the pre-treatment group than that in the control group. *Lachnospiracea_incertae_sedis* was positively associated with CRH concentration (*p* < 0.05). After treatment, *Sutterellaceae* was negatively associated with IL-2 and TNF-α (*p* < 0.05).

**Conclusion:**

This study found that imbalance of the gut microbiota may be involved in the pathogenesis of infantile spasms and is related to the response to adrenocorticotropic hormone (ACTH). *Lachnospiraceae* and *Lachnospiracea_incertae_sedis* might be involved in the disease onset. *Sutterellaceae* might have a link to children’s improved health.

## 1 Introduction

Infantile spasms (IS), also known as West syndrome, is a refractory epilepsy syndrome characterized by clustered nodding, hugging spasm episodes, a hypsarrhythmic pattern in the electroencephalogram (EEG), and neurodevelopmental delay (1). Recently, IS has been reclassified into infantile epileptic spasms syndrome (IESS) to encompass those patients who do not fully meet the criteria for West syndrome (2). This condition primarily affects infants, with an incidence of approximately 2 to 5 per 10,000, predominantly in male infants, and peaks between the ages of 4 and 7 months (3). According to the most recent American Expert Consensus in 2010, adrenocorticotropic hormone (ACTH) and vigabatrin are the most commonly used first-line medications for this condition (4). Other therapeutic approaches, such as glucocorticoids, topiramate, pyridoxine, and a ketogenic diet, are often utilized as initial treatments in resistant or relapsed cases that do not respond to first-line medications (5), although these are generally less effective. It is generally acknowledged that the stress mechanism and the neuroinflammatory mechanism, caused by the disturbance of the hypothalamus–pituitary–adrenal (HPA) axis, are implicated in the pathogenesis of IS (6, 7).

In recent years, there have been increasing studies on the microbiota–gut–brain axis (MGBA) (8, 9), whose mechanism of action involves the gut microbiota being bidirectionally connected to the brain through the relevant pathways of the gut–brain axis (10) and may play a role in the pathogenesis of epilepsy. These pathways include the neuroendocrine (HPA axis) (11), the vagus nerve, the intestinal immune, the neurotransmitter, and the neuromodulator pathways (12, 13). This study focused on how the neuroendocrine and inflammatory response pathways of the gut microbiota affect IS.

The mechanisms driving the progression of IS are not well understood. Therefore, we aimed to investigate the changes and correlations of the gut microbiota, the HPA axis hormones, and the inflammatory cytokines in children with IS before and after treatment in order to provide a reference for future pathogenesis research.

## 2 Materials and methods

### 2.1 Subjects

Children with IS who were hospitalized in the Department of Neurology at our hospital and treated with ACTH from February 2021 to December 2021 were recruited into the case group. All children met the IS diagnostic criteria of the 2010 US Expert Consensus (4, 7). The inclusion criteria for the case group were as follows: 1) children diagnosed for the first time and not previously treated with antiepileptic drugs; 2) those who met the diagnostic criteria for IS established by the 2010 US Expert Consensus; and 3) those aged between 1 and 12 months. The exclusion criteria were: 1) severe malnutrition or excess nutrition; 2) history of digestive diseases and infections in the past 2 weeks with antibiotic treatment; 3) those immunocompromised and those who took hormones or immunosuppressants before diagnosis; 4) those with neurological disorders other than intracranial hemorrhage, stroke, and other epilepsy; and 5) patients with hematological malignancies (such as leukemia) that can cause impaired immunity or intracranial tumors that can disrupt hormone secretion or cause secondary epilepsy. The exclusion criteria after 2 weeks and 1 month of treatment were: 1) not receiving ACTH therapy during treatment; 2) with digestive diseases after enrollment; 3) using antibiotics and probiotics after enrollment; and 4) with neurological disorders other than intracranial hemorrhage, stroke, and other epilepsy. The pre-treatment group was defined as group A, the 2 weeks after treatment group was defined as group B, and the 1 month after treatment group was defined as group C. After 2 weeks of treatment, children with basic remission or with more than 50% decrease of daily seizure occurrence were classified as the effective group according to the clinical relief after using ACTH in the case group. Patients with less than a 50% reduction in daily seizure occurrence, or with little or no remission, were classified as the ineffective group (8).

Healthy children aged 1–12 months were recruited as a blank control group (group D) for the collection of single fecal and blood samples. The technology roadmap is shown in **Supplementary Figure S1**. This study has been reviewed by the ethics committee with number HCHLL-2020-53.

### 2.2 Methods

#### 2.2.1 Sample and general information collection

*Blood samples*: The case group had a first blood collection before treatment, a second blood collection 2 weeks after ACTH treatment, followed by a third collection 1 month after the administration of ACTH or other medications. The samples were then centrifuged after collection, with the upper serum retained and frozen in a refrigerator at −80°C for testing.

*Stool specimen*: Three stool samples were collected before, 2 weeks after, and 1 month after treatment. All fecal samples were collected and immediately frozen in ice boxes before transportation to the laboratory within 30 min and then stored at −80°C.

*Basic information*: The following data were collected: name, gender, age, date of birth, weight, height, gestational age, birth weight, birth method, birth condition and feeding method, clinical data, type of diagnosis, pattern of seizure, frequency of seizures, duration of each seizure, time of the first onset, and EEG results.

*Follow-up of treatment effect*: Data on the current status of medication, the clinical efficacy (seizure frequency), and EEG changes were collected.

*Drug use*: After the diagnosis of IS, all children in the case group were treated with ACTH intravenous fluids and completed a 2-week course of treatment in the first and second weeks of treatment. During weeks 3–4 of treatment, three children continued ACTH therapy, while the remaining children received oral prednisone and other antiepileptic drugs.

#### 2.2.2 Detection of HPA axis hormones and inflammatory cytokines

Cortisol hormone (COR) was measured using a chemiluminescent immunoassay (CLIA). The enzyme-linked immunosorbent assay (ELISA) method was used to measure ACTH and corticotropin-releasing hormone (CRH). The cytometric bead array (CBA) method was used to detect seven cytokines (i.e., IL-2, IL-4, IL-6, IL-10, IL-17α, TNF-α, and IFN-γ).

#### 2.2.3 DNA extraction and high-throughput 16S rDNA gene sequencing

16S rDNA amplicon sequencing was performed by Genesky Biotechnologies Inc. (Shanghai, 201315, China). Briefly, total genomic DNA was extracted using the FastDNA^®^ SPIN Kit for Soil (MP Biomedicals, Santa Ana, CA, USA) according to the manufacturer’s instructions. The integrity of genomic DNA was examined through agarose gel electrophoresis, while its concentration and purity were determined using the NanoDrop 2000 and Qubit 3.0 spectrophotometer. The V4–V5 hypervariable regions of the 16S rDNA gene were amplified with the primers 515F (5'-GTGCCAGCMGCCGCGG-3') and 907R (5'-CCGTCAATTCMTTTR AGTTT-3') and then sequenced using the Illumina MiSeq 6000 platform.

#### 2.2.4 Gut microbial analysis

The raw read sequences were further filtered to remove adapter sequences, primers, and low-quality reads to improve the accuracy of the subsequent analysis.

Alpha diversity was evaluated using abundance and diversity indices. Principal coordinates analysis (PCoA) of beta diversity (based on the Bray–Curtis distance) based on the operational taxonomic unit (OTU) abundance table was performed to evaluate the community composition and structure of the gut microbiota. Linear discriminant analysis effect size (LEfSe) was used to identify the species that are most likely to explain differences between groups with the linear discriminant analysis (LDA) histogram and the cladogram. Metastats analysis compared the samples between groups at different taxonomy levels, which determined the species with significant differences at each taxonomic level. A heat map for Spearman’s rank-sum correlation coefficient was used to evaluate the correlation between the gut microbiota of each group and the levels of inflammatory cytokines and HPA axis hormones.

#### 2.2.5 Statistical analysis

The experimental data were analyzed using SPSS (Version 25) and R software (Version 4.2.3). The measurement data, when normally distributed, were expressed as the mean ± standard deviation (*X* ± *S*), with an independent-samples *t*-test and analysis of variance used to compare differences among groups; otherwise, data were expressed as the median and interquartile range [*M* (*P*_25_–*P*_75_)], with the Mann–Whitney test and the Kruskal–Wallis test used to compare differences among groups. *Post-hoc* multiple tests were used for pairwise comparisons between groups. Categorical data were expressed as percentages, and the comparison differences between two groups were evaluated with the chi-square test. The correlation between the gut microbiota and the levels of inflammatory cytokines and HPA axis hormones was evaluated using Spearman’s rank-sum correlation coefficient. A *p*-value <0.05 was considered as statistically significant.

## 3 Results

### 3.1 Data analysis between the pre-treatment group and the control group

#### 3.1.1 Clinical characteristics of subjects

In this study, 19 children were recruited as cases based on the inclusion criteria, samples from whom were collected before treatment and 2 weeks and 1 month after treatment. During the collection process, two cases were not treated with ACTH, one case was excluded due to infection and antibiotic use during hospitalization, and two cases were lost during follow-up 1 month after treatment. Finally, 14 children with IS were included into the case group. There were 9 children out of the 14 in the case group who had more than three seizures per day, while four children had more than five seizures. Four children had each seizure lasting more than 1 min. There were five children with more than 10 times nodding and/or hugging spasms. There were no significant differences in the general data such as age, gestational age, birth weight, and mode of delivery between the pre-treatment group and the control group (*p* > 0.05), as shown in **Table 1**.

**Table 1:** Clinical characteristics of the subjects in the pre-treatment and control groups.

		Pre-treatment group (*n* = 14)	Control group (*n* = 14)	*p*
Age (months)		6.64 ± 3.15	7.21 ± 2.72	0.61
Gender	Male	7	7	1
	Female	7	7	
Gestational age (weeks)	Mature	14	13	1
	Premature	0	1	
Birth weight (g)	≥2,500	12	13	0.60
	<2,500	2	1	
Delivery	Cesarean section	7	4	0.44
	Normal delivery	7	10	
Feeding	Breastfeeding	6	5	0.79
	Mixed feeding	4	3	
	Formula milk	4	6	
Intrauterine distress	Yes	1	1	1
	No	13	13	
Seizure frequency	≤5/day	10	0	
	>5/day	4	0	
Seizure duration (min)	≤1	10	0	
	>1	4	0	

#### 3.1.2 Comparison of the HPA axis hormone and inflammatory cytokine levels between the two groups

The analysis revealed that the levels of CRH, IL-2, IL-4, IL-6, and IL-17α in the pre-treatment group were significantly higher than those in the control group (*p* < 0.05) (**Table 2**).

**Table 2:** Comparison of the levels of the hypothalamus–pituitary–adrenal (HPA) axis hormones and inflammatory cytokines between the pre-treatment and control groups.

	Pre-treatment group (*n* = 14)	Control group (*n* = 14)	*t*/*z* value	*p*
CRH (ng/ml)	31.50 ± 4.42	19.76 ± 4.05	53.718	0.000**
ACTH (pg/ml)	43.018 (37.4–46.3)	45.172 (44.0–48.2)	−1.149	0.251
COR (g/dl)	12.056 (5.6–18.7)	8.323 (6.5–10.8)	−0.827	0.408
IL-2 (pg/ml)	2.610 (2.375–4.513)	2.045 (1.005–2.738)	−2.183	0.029*
IL-4 (pg/ml)	3.855 (3.003–5.268)	2.370 (1.943–3.153)	−2.918	0.004**
IL-6 (pg/ml)	5.060 (4.353–9.838)	3.620 (2.780–6.393)	−2.184	0.029*
IL-10 (pg/ml)	5.025 (4.160–6.393)	4.180 (3.428–6.385)	−1.264	0.206
IL-17α (pg/ml)	14.565 (10.115–17.925)	8.835 (5.630–10.605)	−2.964	0.003**
TNF-α (pg/ml)	4.400 (3.213–6.245)	3.085 (1.555–5.683)	−1.701	0.089
IFN-γ (pg/ml)	2.180 (0.925–2.698)	1.285 (0.925–1.908)	−1.057	0.29

CRH, corticotropin-releasing hormone; ACTH, adrenocorticotropic hormone; COR, cortisol hormone.

*p < 0.05; **p < 0.01.

#### 3.1.3 Analysis of the gut microbiota differences between the two groups

**Figure 1:**
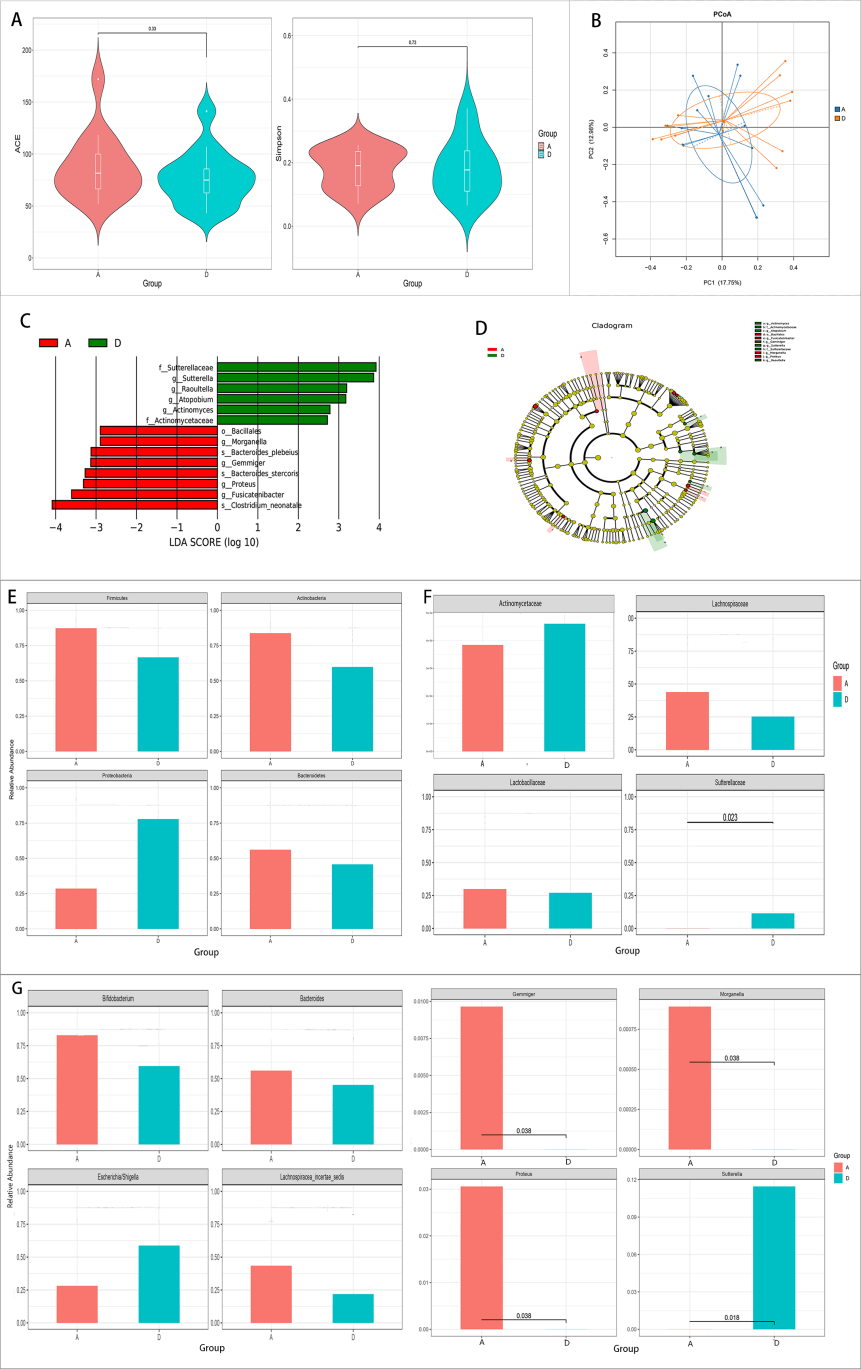
Comparison of the gut microbiota between the pre-treatment group (group A) and the control group (group D). **(A, B)** Comparison of the alpha diversity **(A)** and the beta diversity **(B)** between the two groups. **(C, D)** Linear discriminant analysis (LDA) value distribution and the cladogram of the linear discriminant analysis effect size (LEfSe) between the two groups. **(E–G)** Metastats analysis at the phylum **(E)**, family **(F)**, and genus **(G)** levels between the two groups.

There were no significant differences in the alpha diversity values between the pre-treatment group and the control group (*p* > 0.05). The PCoA found that the gut microbiota composition of the pre-treatment group was highly similar to that of the control group (**Figures 1A, B**).

The results of LEfSe showed that six bacteria were enriched in the control group and eight bacteria were enriched in the pre-treatment group (**Figures 1C, D**).

Metastats analysis was conducted to determine the species with significant differences at the phylum, genus, and family levels. No significant differences were found in the major gut microbiota at the phylum level (**Figure 1E**). However, at the genus and family levels, *Proteus*, *Gemmiger*, and *Morganella* increased, while *Sutterella* and *Sutterellaceae* decreased significantly in the pre-treatment group (*p* < 0.05) (**Figures 1F, G**).

### 3.2 Data analysis of the case group before treatment and 2 weeks and 1 month after treatment

#### 3.2.1 Comparison of the HPA axis hormone and inflammatory cytokine levels among the three groups

The levels of the HPA axis hormones and inflammatory cytokines in the case group decreased after drug therapy. Compared with the pre-treatment group, the levels of CRH and ACTH after 2 weeks of ACTH treatment decreased significantly (*p* < 0.05). After 1 month of treatment with ACTH and other drugs, the levels of CRH, ACTH, and COR decreased significantly compared with those in the pre-treatment group (*p* < 0.05). After 1 month of treatment with ACTH and other drugs, the levels of IL-2, IL-4, IL-6, and IL-17α decreased significantly compared with those before treatment (*p* < 0.05) (**Table 3**).

**Table 3:** Comparison of levels of the hypothalamus–pituitary–adrenal (HPA) axis hormones and inflammatory cytokines in the case group before treatment and 2 weeks and 1 month after treatment.

	Pre-treatment group (*n* = 14)	2 weeks after treatment group (*n* = 14)	1 month after treatment group (*n* = 14)	*t*/*z* value	*p*
CRH (ng/ml)	32.466 (28.919–34.368)^a,c^	22.376 (17.999–26.193)^a^	18.465 (15.943–20.335)^c^	26.359	0.000**
ACTH (pg/ml)	43.018 (37.398–46.273)^a,c^	32.181 (31.005–37.954)^a^	27.226 (23.710–32.948)^c^	19.752	0.000**
COR (µg/dl)	12.056 (5.645–18.727)^c^	8.475 (7.393–10.470)^b^	4.854 (1.168–7.362)^b,c^	10.366	0.006**
IL-2 (pg/ml)	2.610 (2.375–4.513)^c^	1.770 (0.797–3.212)	1.510 (0.902–2.715)^c^	8.099	0.017*
IL-4 (pg/ml)	3.855 (3.002–5.268)^c^	3.035 (1.295–4.772)	1.605 (0.893–3.200)^c^	10.236	0.006**
IL-6 (pg/ml)	5.060 (4.353–9.838)^c^	3.875 (2.367–5.093)	3.160 (1.442–4.232)^c^	11.38	0.003**
IL-10 (pg/ml)	5.025 (4.160–6.393)	3.150 (1.640–4.377)	3.845 (1.440–6.450)	5.546	0.062
IL-17α (pg/ml)	14.565 (10.115–17.925)^c^	10.470 (3.667–15.970)	6.720 (3.515–11.190)^c^	8.049	0.018*
TNF-α (pg/ml)	4.400 (3.212–6.245)	4.150 (1.340–5.152)	2.420 (0.368–5.232)	4.411	0.11
IFN-γ (pg/ml)	1.903 ± 0.899	1.422 ± 0.811	1.214 ± 0.897	2.312	0.113

CRH, corticotropin-releasing hormone; ACTH, adrenocorticotropic hormone; COR, cortisol hormone.

The three groups were compared in pairs (see below) and post-hoc multiple tests were used.

*p < 0.05; **p < 0.01.

a Comparison of the pre-treatment group and the 2 weeks after treatment group (*p* < 0.05).

b Comparison of the 2 weeks after treatment group and the 1 month after treatment group (*p* < 0.05).

c Comparison of the pre-treatment group and the 1 month after treatment group (*p* < 0.05).

#### 3.2.2 Analysis of the gut microbiota differences among the three groups

**Figure 2:**
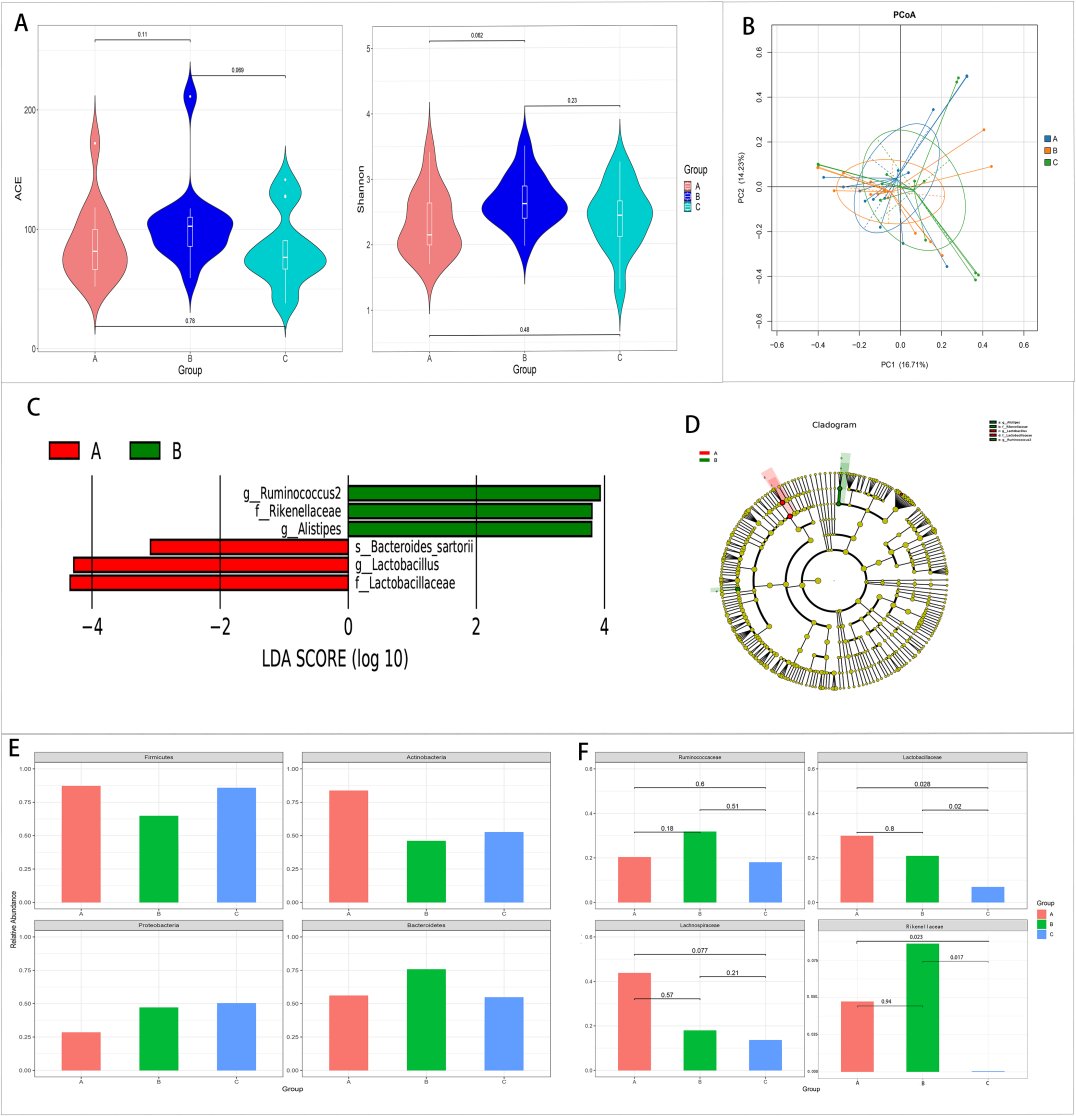
Comparison of the gut microbiota among the pre-treatment (group A), 2 weeks after treatment (group B), and 1 month after treatment (group C) groups in the case group. **(A, B)** Comparison of the alpha diversity **(A)** and the beta diversity **(B)** among the three groups. **(C, D)** Linear discriminant analysis (LDA) value distribution and the cladogram of the linear discriminant analysis effect size (LEfSe) among the three groups. **(E, F)** Metastats analysis at the phylum **(E)** and family **(F)** levels among the three groups.

There were no differences in the alpha and beta diversity among the three groups (**Figures 2A, B**).

The results of LEfSe showed that three bacteria were enriched in the pre-treatment group and three bacteria were enriched in the 2 weeks after treatment group. There was no abundant gut microbiota in the 1 month after treatment group (**Figures 2C, D**).

**Figure 3:**
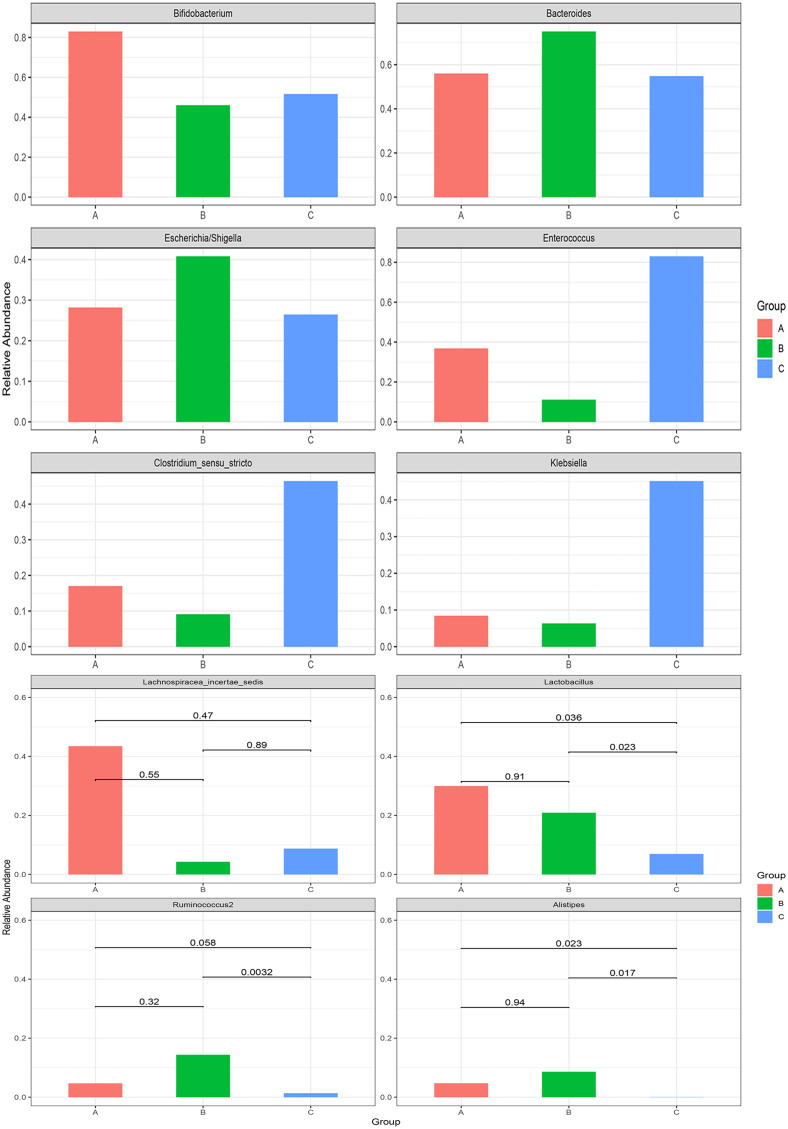
Metastats analysis at the genus level among the pre-treatment group (group A), the 2 weeks after treatment group (group B), and the 1 month after treatment group (group C) in the case group.

Metastats analysis showed that *Lactobacillus* and *Lactobacillaceae* decreased with the extension of the treatment time, and the relative abundance of these two bacteria in the 1 month after treatment group was significantly lower than that in the other two groups (*p* < 0.05). At the same time, *Lachnospiraceae* decreased with the extension of the treatment time. However, no significant difference was found (*p* < 0.05) (**Figures 2E, F**, **3**).

### 3.3 Data analysis between the effective and ineffective groups in the 2 weeks after treatment group

#### 3.3.1 Comparison of the HPA axis hormone and inflammatory cytokine levels between the two groups

The level of IFN-γ was significantly higher and that of CRH was significantly lower in the effective group compared with the ineffective group (**Table 4**).

**Table 4:** Comparison of the levels of the hypothalamus–pituitary–adrenal (HPA) axis hormones and inflammatory cytokines between the effective and ineffective groups.

	Effective group (*n* = 9)	Ineffective group (*n* = 5)	*t*/*z* value	*p*
CRH (ng/ml)	21.16 ± 3.86	25.86 ± 3.02	5.489	0.037*
ACTH (pg/ml)	33.07 ± 3.85	36.85 ± 5.05	2.492	0.14
COR (µg/dl)	9.222 (6.4–10.6)	7.728 (7.6–14.7)	−0.067	0.947
IL-2 (pg/ml)	2.08 ± 1.83	1.81 ± 1.86	0.065	0.803
IL-4 (pg/ml)	3.56 ± 2.76	2.25 ± 1.23	0.997	0.338
IL-6 (pg/ml)	3.980 (2.9–5.2)	2.420 (1.9–11.1)	−0.6	0.549
IL-10 (pg/ml)	3.75 ± 2.40	2.09 ± 1.01	2.109	0.172
IL-17α (pg/ml)	13.810 (4.5–15.2)	4.090 (1.8–10.6)	−1.133	0.257
TNF-α (pg/ml)	3.75 ± 2.73	2.54 ± 1.82	0.771	0.397
IFN-γ (pg/ml)	1.390 (1.2–2.4)	0.590 (0.4–0.8)	−2.467	0.014*

CRH, corticotropin-releasing hormone; ACTH, adrenocorticotropic hormone; COR, cortisol hormone.

*p < 0.05.

#### 3.3.2 Analysis of the gut microbiota differences between the two groups

There were no differences in the alpha and beta diversity between the two groups.

The results of LEfSe revealed three bacteria enriched in the effective group and 10 bacteria enriched in the ineffective group.

**Figure 4:**
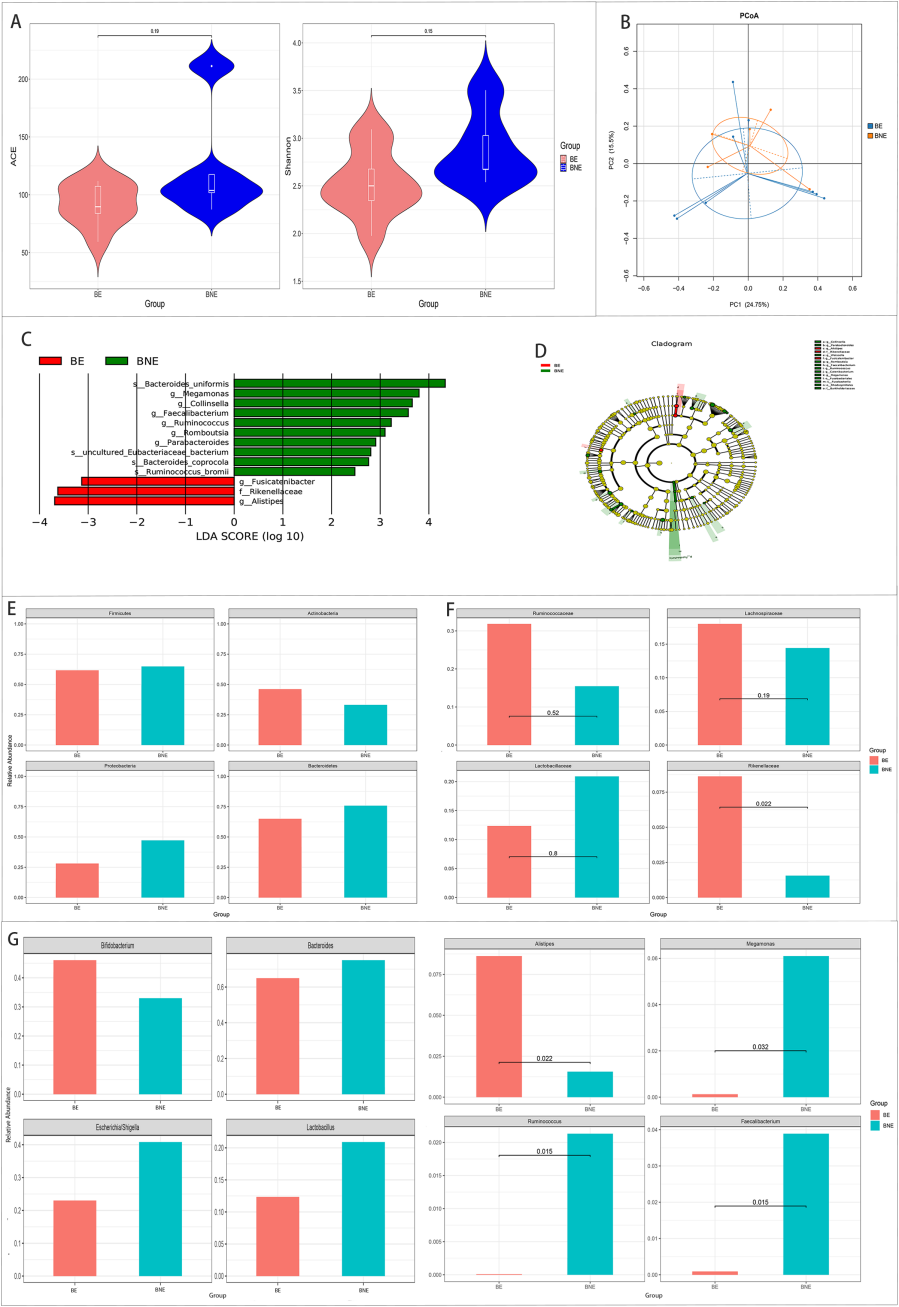
Comparison of the gut microbiota between the effective (BE) and ineffective (BNE) groups in the 2 weeks after treatment group. **(A, B)** Comparison of the alpha diversity **(A)** and the beta diversity **(B)** between the two groups. **(C, D)** Linear discriminant analysis (LDA) value distribution and the cladogram of the linear discriminant analysis effect size (LEfSe) between the two groups. **(E–G)** Metastats analysis at the phylum **(E)**, family **(F)**, and genus **(G)** levels between the two groups.

Metastats analysis showed that *Alistipes* and *Rikenellaceae* were significantly higher and that *Megamonas*, *Faecalibacterium*, and *Ruminococcus* were significantly lower in the effective group than those in the ineffective group (*p* < 0.05) (**Figure 4**).

### 3.4 Correlation analysis between the gut microbiota and the HPA axis hormone and inflammatory cytokine levels

**Figure 5:**
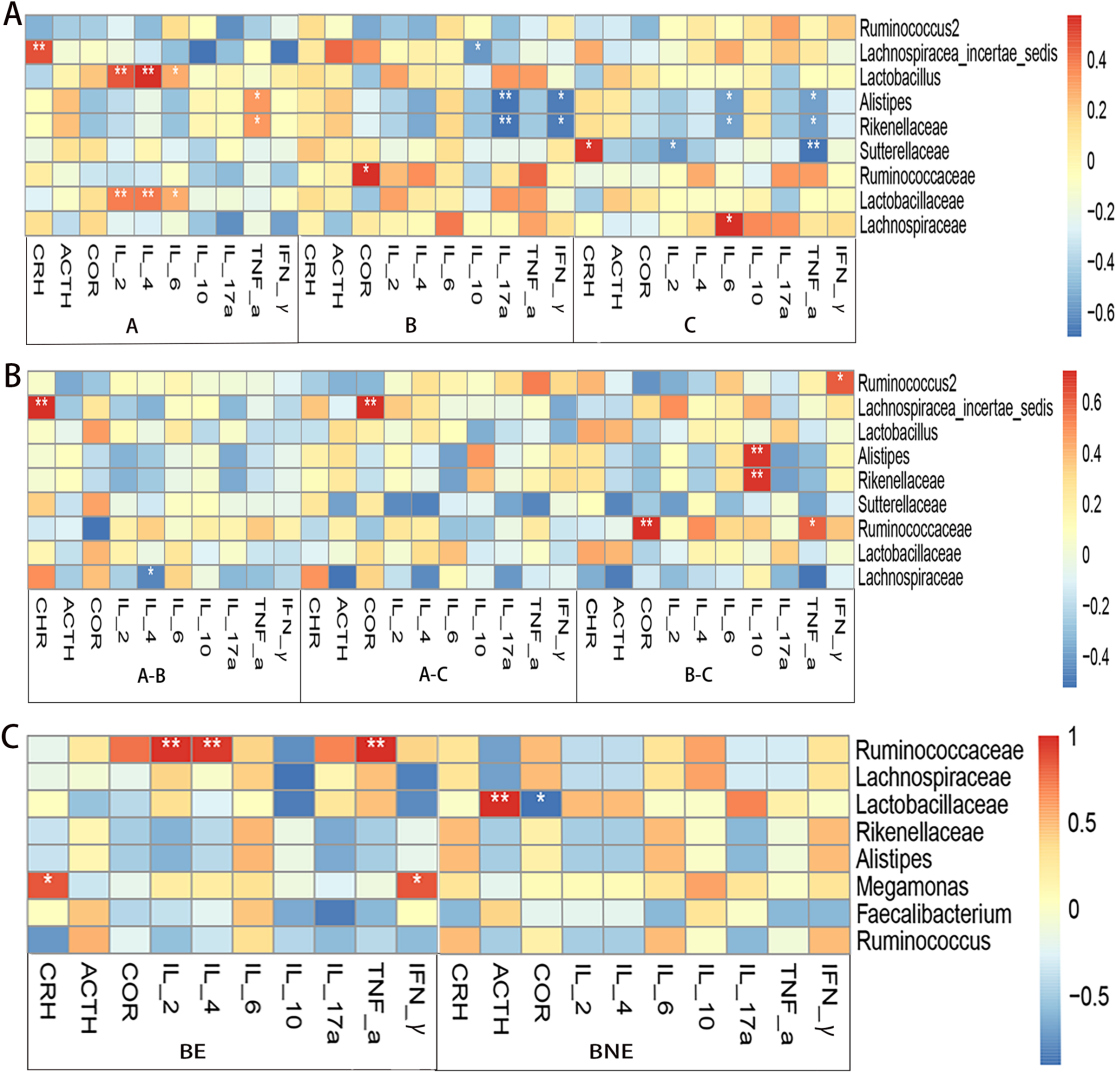
Correlation analysis between the gut microbiota and the levels of hypothalamus–pituitary–adrenal (HPA) axis hormones and inflammatory cytokines. **(A)** Correlation analysis of the pre-treatment (group A), 2 weeks after treatment (group B), and 1 month after treatment (group C) groups in the case group. **(B)** Correlation analysis between the differences in the gut microbiota with differences in the levels of HPA axis hormones and inflammatory cytokines in the case group. **(C)** Correlation analysis of the effective (*BE*) and ineffective (*BNE*) groups *p < 0.05; **p < 0.01.

We used the heat map of the Spearman’s rank-sum correlation coefficients to determine the correlation between the gut microbiota and the HPA axis hormone and inflammatory cytokine levels. In the pre-treatment group, *Lachnospiracea_incertae_sedis* was positively associated with CRH. *Lactobacillaceae* and *Lactobacillus* were positively associated with IL-2 and IL-4. *Alistipes* and *Rikenellaceae* were negatively correlated with IL-17a and IFN-γ in the 2 weeks after treatment group. In the 1 month after treatment group, *Sutterellaceae* was positively associated with CRH and negatively associated with IL-2 and TNF-α. *Alistipes* and *Rikenellaceae* were negatively associated with IL-6 and IFN-α (**Figure 5A**).

Subsequently, we compared the correlation between the differences in the gut microbiota and the differences in the levels of HPA axis hormones and inflammatory cytokines. It was found that *Lachnospiracea_incertae_sedis* was positively associated with CRH and COR*. Alistipes* and *Rikenellaceae* were negatively associated with IL-10 (the relative abundance of *Alistipes* and *Rikenellaceae* in group B was higher than that in group C, while the level of IL-10 in group B was lower than that in group C) (**Figure 5B**).

In the effective group, *Ruminococcaceae* was positively associated with IL-2, IL-4, and IFN-γ. *Lactobacillaceae* was positively associated with ACTH (**Figure 5C**).

## 4 Discussion

In this study, it was found that the levels of IL-2, IL-4, IL-6, and IL-17α in the pre-treatment group were considerably greater than those in the control group, indicating that the level of inflammatory cytokines in infants with IS increased. After disruption of the central nervous system in infants with spasm, damage of the endothelial cells in the brain tissue causes the release of a variety of inflammatory factors. Based on pathological research, the inflammatory regulators and receptors have been found to be greatly enhanced in brain tissue specimens from patients with epilepsy, viral encephalitis, and other neurological injuries, as well as in the cerebral cortex and limbic tissues of rats with epilepsy. For instance, inflammatory factors such as IL-2 and IL-6 in serum showed a substantial increase (14, 15). IL-2, a γc family cytokine, is primarily secreted by T cells, promoting lymphocyte proliferation, inducing natural killer (NK) cells, and participating in immune regulation (16). In the central nervous system, IL-6 possesses a range of biological actions, which can regulate the functions of nerve cells (17). Studies have indicated that IL-6 is overexpressed in the brain tissue of patients with epilepsy and is released into circulation through cerebral blood vessels. The level of IL-6 was found to be much higher in patients with recurrent generalized tonic–clonic seizures compared with those with single seizures (18). IL-17α is an important inflammatory factor that can stimulate macrophages, endothelial cells, fibroblasts, and epithelial cells to produce a variety of inflammatory factors, promoting the incidence and development of neurological diseases (19). Related studies have reported that the level of IL-17α was higher in the cerebrospinal fluid of children with acute seizures, which is consistent with our findings (20).

This study discovered that the CRH level in the pre-treatment group was much greater than that in the control group, and the levels of CRH, ACTH, and COR on the HPA axis decreased following ACTH therapy. Previous studies have shown that the concentrations of CRH can increase in response to certain conditions such as external stress and can trigger IS by interacting with brain-specific cortical targets (21). ACTH, an anti-epileptic drug, is currently derived from corticotropin extracted from exogenous bovine, horse, and other animals. Its mechanism may involve negative feedback to inhibit CRH secretion in the HPA axis, thereby temporarily suppressing the secretion of endogenous ACTH, resulting in a decreased ACTH concentration after treatment (22). COR is a part of the HPA axis, regulated by superiors. Most of the children in this study received prednisone oral therapy following ACTH administration at the end of 2 weeks of treatment. Prednisone, acting as COR, could regulate the upstream CRH and ACTH levels with negative feedback, leading to a decrease in the COR concentration after 1 month of treatment. According to previous studies, patients with epilepsy had abnormal immune function, which affected the endocrine system through the “neuroendocrine–immune network” and promoted cortisol release (23).

Interestingly, the levels of IL-2, IL-4, IL-6, and IL-17α decreased significantly after treatment, which further explained the effect of cranial nerve injury and inflammatory response on the pathological changes of IS. Previous studies have demonstrated that ACTH exerts a regulatory effect on the immune-mediated inflammatory responses (24). This mechanism might account for the reduction in the levels of inflammatory cytokines in the case group following treatment. Given the incomplete understanding of the specific pathogenesis of IS, additional research is needed to elucidate the precise mechanism of ACTH in the treatment of IS.

There were no significant differences in the gut microbiota diversity across all groups in this study, suggesting that the gut microbiota of patients were similar to those of normal infants. Moreover, the treatment and efficacy had little effect on the diversity of the gut microbiota, which is consistent with the findings of a study on infantile spasmodic gut microbiota (9).

Compared with the control group, *Sutterellaceae* and *Sutterella* decreased significantly in the pre-treatment group. *Sutterella* belongs to *Sutterellaceae*, and animal model studies have shown that *Sutterellaceae* are typical intestinal dominant bacteria whose variations in abundance are related to the function of the intestinal mucosal barrier (25). These findings showed that a reduction in *Sutterellaceae* is associated with disease severity.

When comparing the conditions before and after treatment in the case group, *Lachnospiraceae* was found to decrease with the extension of the treatment time. After 2 weeks of treatment, *Lachnospiracea_incertae_sedis* decreased compared with that before treatment. However, there was no significant difference. *Lachnospiracea_incertae_sedis*, which belongs to *Lachnospiraceae*, are anaerobic bacteria known to promote glucose fermentation and to produce lactic and formic acids, impacting intestinal permeability and stimulating intestinal chromaffin cell synthesis and the release of serotonin (5-HT) (26). 5-HT, a neurotransmitter, influences the excitability/inhibitory balance of the cerebral cortex and subcortical regions and is involved in various physiological and pathological processes in the brain. Studies have shown that 5-HT could affect the onset and progression of epilepsy by influencing the response to ACTH therapy in infants with IS (27). Furthermore, other research found that the relative abundance of *Lachnospiracea_incertae_sedis* in patients with Parkinson’s disease increased, while it decreased significantly in patients who responded to treatment (28). This supports the notion that, as therapy continued and the condition improved, the dominant bacteria of the gut microbiota in children with IS gradually shifted toward those of a normal human gut microbiota.

After 2 weeks of treatment in the case group, *Alistipes* and *Rikenellaceae* increased in the effective group, while *Megamonas*, *Faecalibacterium*, *Ruminococcus*, and *Romboutsia* increased in the ineffective group. *Ruminococcus* belongs to *Firmicutes*, and its abundance increased in the gut microbiota of drug-resistant epilepsy patients (29). *Ruminococcus* and other uncommon bacteria could participate in the occurrence of epilepsy by regulating the adenosine triphosphate binding cassette transporters (29). *Ruminococcus* has been shown to be positively correlated with glutamate and to be negatively correlated with 5-HT in animal studies (30). In addition, *Ruminococcus* is associated with lower levels of *N*-acetylaspartic acid in patients with epilepsy, which is a neuronal health marker (31). Therefore, *Ruminococcus* is thought to influence drug susceptibility to epilepsy by regulating certain neurotransmitters.

This study found that *Lachnospiracea_incertae_disedis* was positively associated with CRH. *Lachnospiracea_incertae_disedis* is significantly higher in children with autism spectrum disorder, and studies have shown that autism is associated with disorders of the HPA axis caused by maternal stress and trauma during pregnancy (32). These might suggest that *Lachnospiracea*_*incertae_disedis* could influence the occurrence of neurological diseases through the HPA pathway. *Ruminococcaceae* was positively associated with IL-2, IL-4, and IFN-γ. This is consistent with the increase in the relative abundance of *Ruminococcus* in the ineffective group.

It was also discovered that, after treatment, *Sutterellaceae* was positively associated with CRH and negatively associated with IL-2 and TNF-α. *Sutterella* has been shown to be negatively associated with inflammatory cytokines (IL-12, IL-13, and IFN-γ) in a clinical cohort study (33), which is consistent with this study. The CRH levels increased and *Sutterellaceae* decreased, which did not match the expected results. This could be related to factors such as the experimental design, the subjects’ age, and the sample acquisition time, among others, which made it difficult to cross-compare some experiments.

In summary, the MGBA plays an important role in IS. In a study on the association between IS and the gut microbiota, researchers found that *Lactobacillus*, *Roseburia*, and *Lachnospira* were lower in the IS group than those in the healthy group. Compared with that in the ACTH-NR (no response) group, *Bifidobacterium* was higher in the ACTH-response group (34). Studies have shown that probiotic supplementation could significantly reduce the frequency and the severity of seizures (35, 36). In addition, research found that the ratio of *Bacteroidota-to-Firmicutes* increased in epileptic (Epi) rats compared with non-epileptic (No-Epi) and sham control rats (37). These are consistent with the findings of this study, where the gut microbiota was involved in the development of IS. Specific gut microbiota could be used as a potential therapeutic target for IS, and disease control might be achieved by restoring the gut microbiota.

This study has certain limitations. Firstly, the sample size was small, which should be expanded in future studies to improve the accuracy of the results. Secondly, in this study, 16S rDNA sequencing was used for the gut microbiota, which could only ensure the accuracy of the microflora at the genus level and above. Therefore, future research needs to use metagenomic sequencing technology to improve the breadth and accuracy of sequencing. Thirdly, the follow-up time of the study was short, and follow-up should be up to 3 and 6 months and 1 year after treatment. Data on the improvement of the children, the serological samples at the time of review, and the stool results should be collected in order to track the changes in the relationship of the HPA hormones, inflammatory cytokines, and gut microbiota. Finally, we studied the relationship of the HPA axis hormones, inflammatory cytokines, and gut microbiota; however, the various ways how the gut microbiota affects the development of IS, as well as the in-depth mechanism of this effect, have not been studied. Therefore, future studies need to conduct animal and cell experiments to explore how the gut microbiota affects the pathogenesis of IS.

## 5 Conclusion

The gut microbiota of children with IS differed from that of healthy children. *Lachnospiraceae* and *Lachnospiracea_incertae_disedis* might be associated with the disease onset. *Sutterellaceae* might be linked to children’s improved health. In addition, certain gut microbiota might affect the levels of some HPA axis hormones or inflammatory cytokines in IS.

## Data Availability

The datasets presented in this study can be found in online repositories. The names of the repository/repositories and accession number(s) can be found https://www.ncbi.nlm.nih.gov/sra/PRJNA1145298, PRJNA1145298.
