# Supplementary figures and images for: Study on the gut microbiota, HPA, and cytokine levels in infantile spasms
Source: Front Immunol. 2024 Oct 10;15:1442677. doi: 10.3389/fimmu.2024.1442677 (PMC11499101; doi:10.3389/fimmu.2024.1442677)

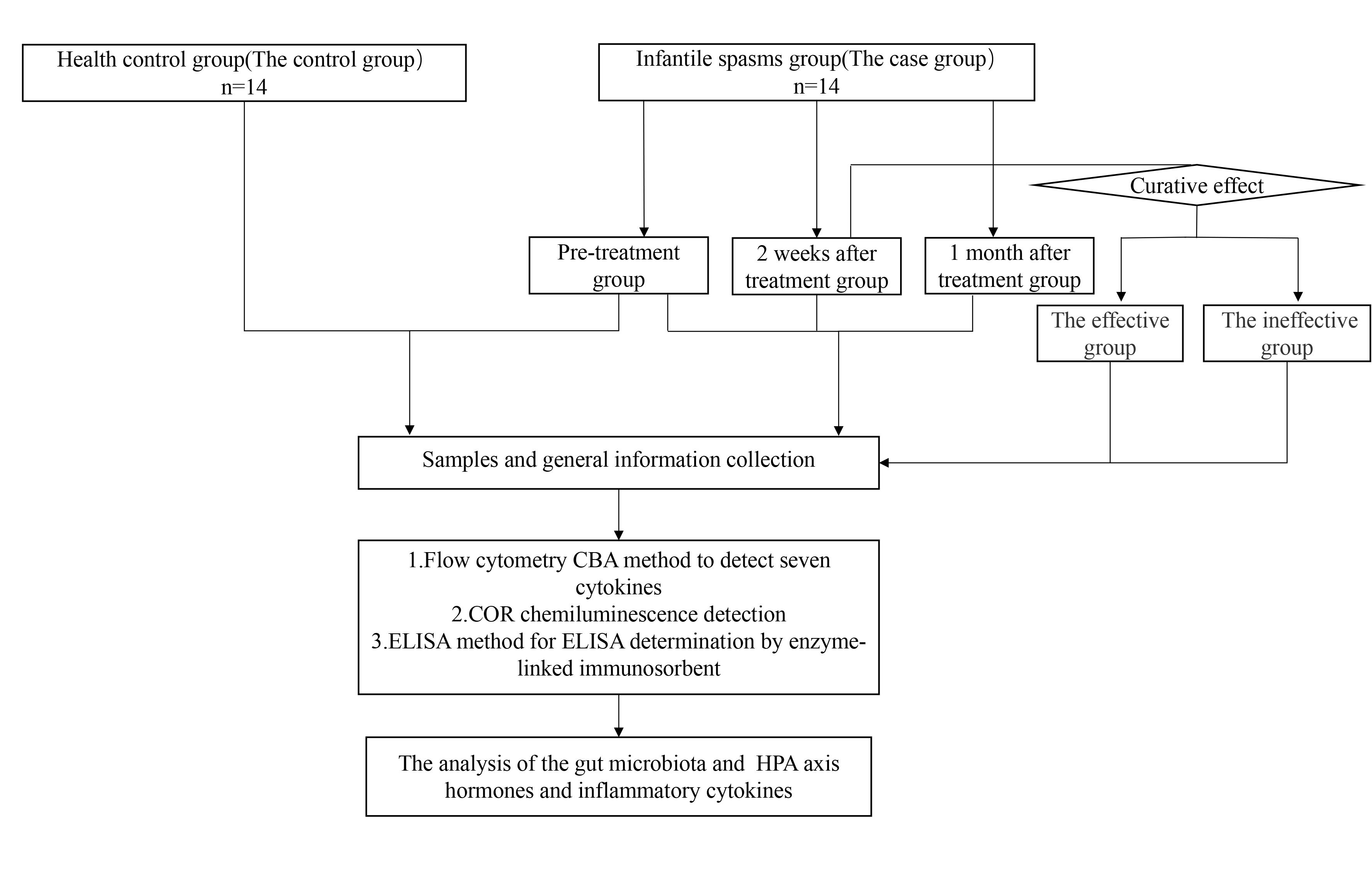

Supplement: Supplementary file 1 [file Image1.tif]
